# Aspergillus Fungemia Versus Pseudofungemia: A Case Series Illustrating Diagnostic Criteria, Risk Factors, and Clinical Implications

**DOI:** 10.7759/cureus.111608

**Published:** 2026-06-27

**Authors:** Devi B Yalamanchili, Sreenath Varma, Shivan Shah

**Affiliations:** 1 Department of Oncologic Sciences, University of South Florida Morsani College of Medicine, Tampa, USA; 2 Department of Internal and Hospital Medicine, Moffitt Cancer Center, Tampa, USA; 3 Department of Internal Medicine, Division of Infectious Diseases and International Medicine, University of South Florida, Tampa, USA

**Keywords:** aspergillus, aspergillus fungemia, aspergillus terreus, invasive aspergillosis, pseudofungemia

## Abstract

*Aspergillus* species are environmental fungi that can cause invasive disease in susceptible hosts. Detection in blood cultures is uncommon and may represent true fungemia or pseudofungemia. Previously, *Aspergillus* fungemia was primarily described in patients with hematologic malignancies and transplant recipients, but it is becoming a more common cause of infection as diverse at-risk patient populations emerge. In this case series, we describe two patients with positive blood cultures for *Aspergillus*. The first is a 52-year-old man with metastatic colorectal cancer and multiple risk factors who developed confirmed *Aspergillus terreus *fungemia. The second is a 77-year-old man with chronic myelomonocytic leukemia following allogeneic stem cell transplantation in whom a single blood culture yielded *Aspergillus* without clinical evidence of invasive infection, consistent with pseudofungemia. Distinguishing true fungemia from contamination is critical. Corticosteroid use, intravascular devices, and environmental exposures may predispose even non-neutropenic individuals to invasive disease. Early recognition and targeted antifungal therapy are essential, given the significant associated mortality.

## Introduction

Fungi within the genus *Aspergillus *are widely present in the environment and function as opportunistic pathogens when host defenses are impaired. Invasive disease is most often observed in patients with hematologic malignancies, transplant recipients, or those receiving immunosuppressive therapies [1,2]. Despite this, bloodstream infection due to *Aspergillus* remains a rare finding [1,2]. Cultures yielding *Aspergillus *species may reflect either true infection or contamination [1,2]. The growing use of immunomodulatory and immunosuppressive therapies has broadened the population at risk. Among these, corticosteroids play a prominent role by altering innate immune pathways, including disruption of neutrophil function and reduced macrophage-mediated clearance [3,4]. As the use of immunosuppressive and immunomodulatory therapies has grown, so has reliance on central venous catheters for their administration, creating an additional nidus for infection [1].

Moffitt Cancer Center’s Data Quality Operations and Clinical Systems teams assisted in reviewing all available medical records at Moffitt Cancer Center for blood culture results containing the word “*Aspergillus*” without applying any filters. Only four charts met these criteria. This search was limited by the available records uploaded to PowerChart in Cerner, which was integrated with Moffitt Cancer Center in 2011 [5]. Of these four charts, one was a laboratory-only patient with no accompanying clinical notes available for further review. In another case, the positive culture could not be verified when the medical record number was searched in PowerChart in Cerner. This may have been because the positive culture was obtained before full integration with Cerner. Ultimately, only two patients with verifiable positive blood cultures for *Aspergillus *were identified for inclusion in this case series.

We present two cases of *Aspergillus*-positive blood cultures, one consistent with true fungemia and the other with suspected pseudofungemia. We discuss these cases to investigate risk factors and identify features that distinguish true fungemia from probable contamination. Accurate differentiation is paramount for guiding appropriate management, ensuring timely treatment when bloodstream infection is present while avoiding unnecessary prolonged antifungal therapy when it is not.

## Case presentation

Case 1

A 52-year-old man with sigmoid colon adenocarcinoma metastatic to the liver and lungs presented with progressive dyspnea, fatigue, and intermittent fevers. His last documented fever was 100.8°F (38.2°C), occurring three days before admission. Two peripheral blood cultures were obtained from different sites. His oncologic course included multiple chemotherapy regimens with ongoing disease progression, most recently trifluridine/tipiracil more than one month before admission. In addition, he had been taking dexamethasone 4 mg daily for 10 months for chronic back pain, which was tapered off two weeks before presentation because of iatrogenic Cushing syndrome.

On admission (vital signs and pertinent clinical data are shown in Table 1), his oxygen saturation was 94% on room air. Physical examination was notable for cushingoid facies and chronic abdominal tenderness to palpation related to his colon cancer. CT angiography (CTA) of the chest demonstrated suspected progression of metastatic disease with multiple pulmonary nodules and bulky enlarged mediastinal lymph nodes (Figures 1, 2). Chest CTA also revealed multiple nonocclusive pulmonary emboli (Figure 3). Anticoagulation was initiated. The initial infectious workup was negative, including no growth of pathogens in the peripheral blood cultures, and the patient was discharged home. Four days after discharge, both peripheral blood cultures yielded *Aspergillus*, later identified as *Aspergillus terreus *complex (Figures 4, 5). The patient was immediately contacted and asked to return to the hospital.

**Table 1:** Clinical and laboratory data for Case 1 and Case 2 Normal reference values obtained from PowerChart in Cerner.

Category	Variable	Case 1 - initial admission	Case 1 - readmission	Case 2 - admission	Reference range
Characteristics	Age	52 years	-	77 years	-
	Sex	Male	-	Male	-
	Malignancy	Metastatic sigmoid colon adenocarcinoma	-	Chronic myelomonocytic leukemia	-
	Line access	Implanted port + peripheral intravenous catheter	-	Peripheral intravenous catheter	-
Vitals	Temperature (°C)	37.3 (maximum 38.2 at home)	37.1	36.3	36.5-37.3
	Heart rate (beats/minute)	100	75	77	60-100
	Respiratory rate (breaths/minute)	15	16	18	12-20
	Blood pressure (mmHg)	133/81	117/70	104/68	90/60-120/80
	Oxygen saturation (%)	94 (room air)	95 (room air)	100 (room air)	95-100
Immunosuppression	Medications	Dexamethasone 4 mg daily long term, tapered off two weeks prior	Dexamethasone 1 mg daily	Sirolimus 1 mg five days/week	-
		Trifluridine/tipiracil (>1 month prior)		Prednisone 15 mg daily	-
				Tacrolimus 0.5 mg twice daily	-
				Leflunomide 20 mg daily	-
Hemogram	WBC (k/µL)	9.05	10.18	0.94 ↓	4-10.9
	Absolute neutrophil count (k/µL)	7.05	7.93	0.61 ↓	1.8-7.8
	Hemoglobin (g/dL)	11.9 ↓	11.8 ↓	10.6 ↓	13.4-16.9
	Hematocrit (%)	37.2 ↓	37.2 ↓	32 ↓	40-48
	Platelets (k/µL)	385 ↑	321	12 ↓↓↓	143-382
Infectious/inflammatory markers	Lactate (mmol/L)	-	1.4	-	0.5-2
	Procalcitonin (ng/mL)	-	0.09 ↑	-	0-0.07
Drug levels	Sirolimus (ng/mL)	-	-	>30 ↑	5-14
	Tacrolimus (ng/mL)	-	-	5.9	5-15
Virology	BK virus (copies/mL)	-	-	2380 ↑	Undetected
	Cytomegalovirus (copies/mL)	-	-	5050 ↑	Undetected
Fungal markers	Galactomannan	5.15 (bronchoalveolar lavage, two months prior) ↑	-	Serum negative	Negative
Microbiology	Blood cultures	Two peripheral: *Aspergillus terreus*	Peripheral + implanted port: *A. terreus*	1/2 peripheral: 1 colony of filamentous fungi (*Aspergillus*, non-*fumigatus*)	-
		-	Repeat peripheral + implanted port cultures prior to discharge (on treatment): no growth	Repeat two peripheral cultures: no growth	-

**Figure 1:**
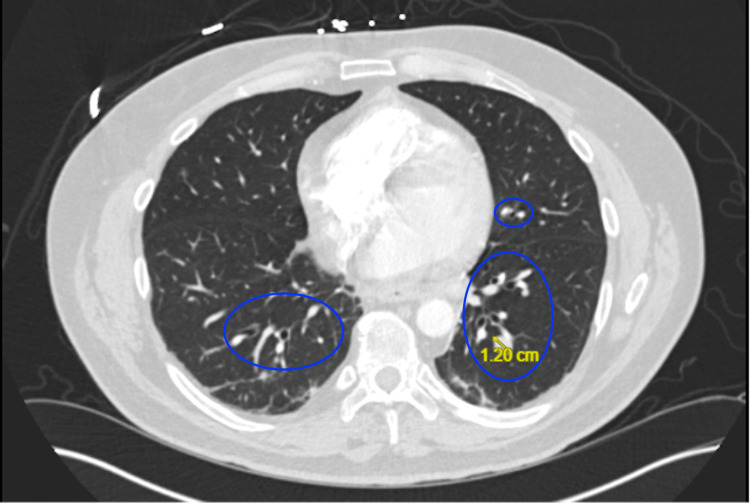
Case 1: CTA chest axial view on initial admission demonstrating multiple pulmonary nodules (blue circles) The largest pulmonary nodule is located in the left lower lobe and measures 1.20 cm (yellow arrow). CTA: CT angiography

**Figure 2:**
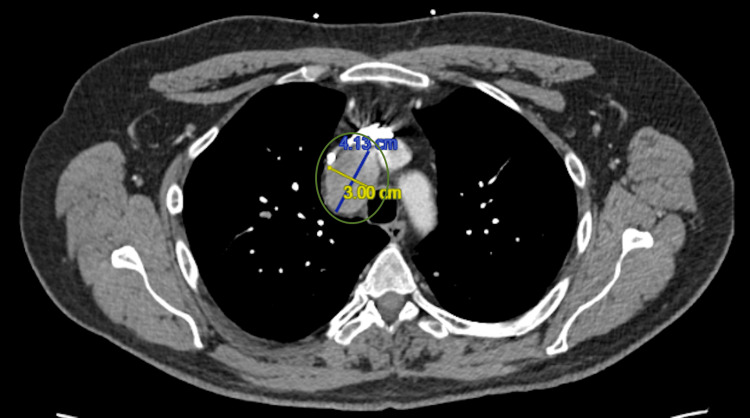
Case 1: CTA chest axial view on initial admission demonstrating an enlarged pretracheal lymph node (green circle) measuring 3.00 cm (yellow arrow) × 4.13 cm (blue line) CTA: CT angiography

**Figure 3:**
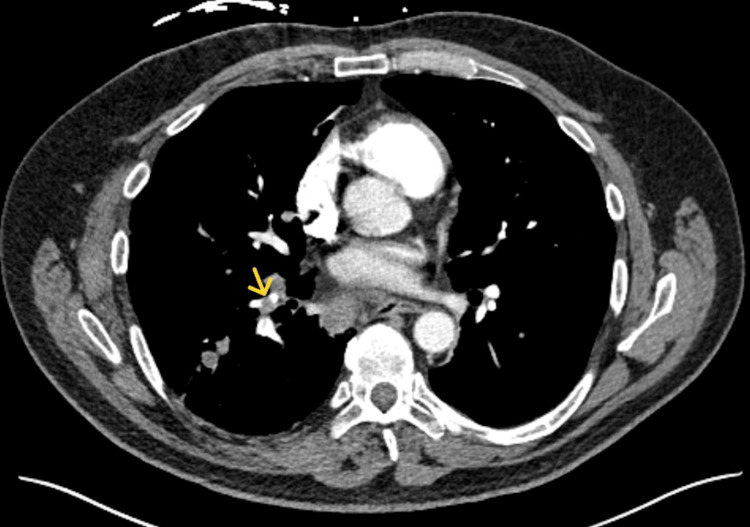
Case 1: CTA chest axial view on initial admission demonstrating a right middle lobe segmental pulmonary embolus (yellow arrow) CTA: CT angiography

**Figure 4:**
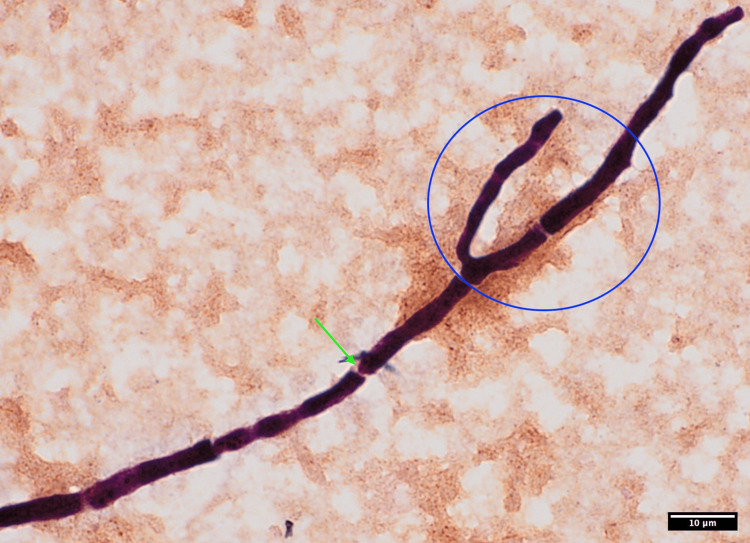
Case 1: Gram stain of the initial aerobic blood culture This stain displays septate hyphae with 45-degree acute-angle branching (blue circle) and regularly spaced septations (green arrow), characteristic of *Aspergillus* species (100x magnification). Scale bar: 10 µm.

**Figure 5:**
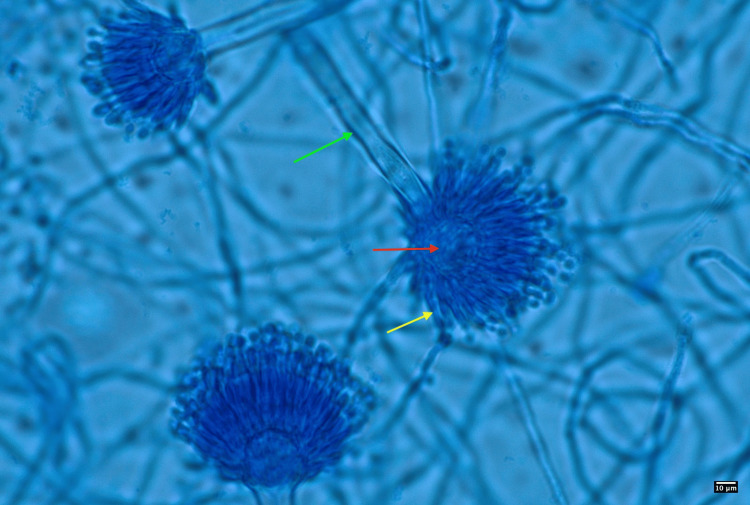
Case 1: LPCB preparation of the initial aerobic blood culture This preparation demonstrates a conidiophore (green arrow) terminating in a vesicle (red arrow) with radiating conidial chains (yellow arrow), typical of *Aspergillus* species morphology (40× magnification). Scale bar: 10 µm. LPCB: lactophenol cotton blue

Upon readmission, repeat cultures, one peripheral and one from the implanted port, were obtained. Infectious Disease was consulted, and liposomal amphotericin B and voriconazole were initiated. Both the peripheral and implanted port cultures later returned positive for *A. terreus*. The implanted port was removed, and the catheter tip was cultured, which also grew *A. terreus.* After further discussion with the patient, he revealed that he had been using his hot tub almost daily to help with his back pain. Further review of the medical record revealed that the patient had also had a positive galactomannan antigen result obtained from bronchoalveolar lavage two months before these admissions, with an elevated galactomannan index of 5.15.

The hospital course was complicated by acute kidney injury, likely due to liposomal amphotericin B, with a peak creatinine level of 3.9 mg/dL, necessitating discontinuation of the drug. He was subsequently transitioned to voriconazole monotherapy. Transthoracic and transesophageal echocardiography showed no evidence of endocarditis. CT of the sinuses did not demonstrate evidence of invasive fungal disease. Follow-up blood cultures remained negative for pathogens. The patient completed a three-month course of voriconazole. During the chart review conducted for the preparation of this case series, it was determined that the patient had died a few months later. There was no evidence that persistent fungal infection contributed to his death. Therefore, he most likely succumbed to his progressive malignancy.

Case 2

A 77-year-old man with chronic myelomonocytic leukemia, a history of allogeneic hematopoietic stem cell transplantation two months before admission, hyperacute cutaneous graft-versus-host disease, and BK virus (human polyomavirus 1) reactivation was admitted for cytomegalovirus reactivation with concern for cytomegalovirus enteritis versus gastrointestinal graft-versus-host disease. The patient was taking sirolimus 1 mg five times weekly and tacrolimus 0.5 mg twice daily for post-transplant immunosuppression. For cutaneous graft-versus-host disease, he was receiving a prednisone taper and was taking 15 mg daily at the time of admission. Vital signs and pertinent clinical data are listed in Table 1. Physical examination was unremarkable, and he did not have reproducible abdominal tenderness to palpation. He was pancytopenic on admission, with a white blood cell count of 0.94 k/µL, an absolute neutrophil count of 0.61 k/µL, an absolute lymphocyte count of 0.14 k/µL, a hemoglobin level of 10.6 g/dL, and a platelet count of 12 k/µL. The sirolimus level was initially greater than 30 ng/mL on admission, so the medication was briefly withheld and then restarted at a lower dose once the level reached the therapeutic range. Cytomegalovirus PCR showed a peak of 5050 copies/mL, with worsening abdominal cramps and spasms before admission.

Endoscopy with biopsy was performed and later returned negative for cytomegalovirus but showed patchy antral erythema and a diminutive gastric ulcer. Biopsies confirmed grade 3 of 4 graft-versus-host disease in the stomach, grade 2-3 of 4 in the duodenum, and grade 2 of 4 in the rectum. He was able to tolerate meals without nausea, vomiting, or diarrhea, and his abdominal cramps resolved; therefore, he was treated supportively for graft-versus-host disease. He was treated with foscarnet for cytomegalovirus reactivation in case it was contributing to his abdominal symptoms. BK virus testing demonstrated 2380 copies/mL (3.4 log₁₀) in serum, associated with lower abdominal spasms and dysuria, without hematuria. He continued on leflunomide for BK virus reactivation. During hospitalization, a single peripheral blood culture yielded one colony of filamentous fungi identified as *Aspergillus *species (non-*fumigatus*). Growth was limited to a single plate. The patient was already receiving voriconazole prophylaxis. Repeat peripheral blood cultures remained negative. No clinical evidence supported invasive aspergillosis, no escalation of antifungal therapy was documented, and the patient appeared to do well without reported complications. No imaging was performed during this admission. He was discharged home with ongoing daily foscarnet infusions at the infusion center and continued tacrolimus, sirolimus, prednisone, and leflunomide, as well as prophylactic voriconazole and pentamidine.

## Discussion

Prior literature indicates that confirmed cases of *Aspergillus* fungemia are sparsely documented, accounting for less than 4% of all fungemias, with a comparable number of reported pseudofungemia events [1,2]. Kaur and de St. Maurice identified only seven cases of catheter-associated *Aspergillus *fungemia through an extensive PubMed search, with their own case constituting the eighth [1]. In one review spanning a 23-year period of 1,453 patients who had undergone hematopoietic stem cell transplantation, only one case of genuine *Aspergillus *fungemia was identified [1]. Even in invasive aspergillosis, fungemia is rare, occurring in approximately 0.1-9.3% of cases [6,7]. *Aspergillus fumigatus* has been reported to account for approximately 75% of *Aspergillus*-positive blood cultures, making the *A. terreus *fungemia in Case 1 even more unusual [2,6].

In Case 1, the pulmonary nodules identified on chest CTA, which were attributed at the time to pulmonary metastases, could have represented pulmonary aspergillosis, given the elevated bronchoalveolar lavage galactomannan index of 5.15 obtained two months before admission. This elevated galactomannan level, which met the criteria for probable invasive aspergillosis according to the Infectious Diseases Society of America (IDSA) guidelines [8], was not acted upon at the time. Although the clinical reasoning behind this decision was not documented, it highlights the diagnostic challenge posed by overlapping malignant and infectious pulmonary findings. Interventional radiology was consulted to biopsy a lung nodule to confirm suspected metastasis; however, a liver biopsy was recommended instead because of concern about the higher procedural risk in the setting of pulmonary emboli. The liver biopsy was positive for colon adenocarcinoma.

The pulmonary emboli, which were attributed to a hypercoagulable state in the setting of progressive malignancy, may have represented thromboses associated with *Aspergillus* infection. Research has shown that *Aspergillus *hyphae invade endothelial cells in both arterial and venous blood vessels through endocytosis, leading to thrombus formation and distal infarction [1,2]. This mechanism may also explain why *Aspergillus *is so rarely recovered from blood cultures, as vascular invasion reduces blood flow through infected vessels [2].

Indicators of true fungemia include reproducible growth in multiple cultures, isolation from intravascular devices, concordant clinical features, and response to antifungal therapy [2]. In Case 1, the patient had fever, associated pulmonary emboli, dyspnea, pulmonary nodules, and an elevated galactomannan index consistent with pulmonary aspergillosis. In addition, his initial two cultures grew *A. terreus* from two different peripheral sites. Repeat peripheral and implanted port cultures, as well as the catheter tip culture, also grew *A. terreus*. He therefore met multiple criteria for true fungemia.

Prolonged neutropenia remains a well-established risk factor for fungemia [2,6]. However, additional contributors are increasingly being recognized, including corticosteroid exposure, intravascular catheters, and environmental exposures (e.g., hot tubs), all of which were present in Case 1 [2,4,6,9]. In a prospective study by Tala-Ighil et al., 93% of patients with mold-positive blood cultures had a central venous catheter [6]. Corticosteroid exposure was noted in 22.5% of patients and was found to be an independent predictor of adverse outcomes (odds ratio, 7.85) [6].

The environmental exposure history in Case 1, specifically, near-daily hot tub use, adds a clinically important yet underappreciated contributor to infection risk. Hot tubs and recreational water sources are recognized reservoirs for opportunistic pathogens capable of causing pulmonary disease through aerosolization and inhalation [9]. Hot tub use in an immunocompromised host represents an environmental risk that warrants explicit counseling. The timeline in Case 1 is consistent with pulmonary infection preceding hematogenous dissemination, as evidenced by the elevated galactomannan index two months before admission for fungemia.

Glucocorticoids impair host defenses through multiple mechanisms, including altered leukocyte trafficking and reduced phagocytic function, affecting almost every immune cell type. This not only impairs the response to infection but may also delay recognition of infection [3,4,10]. Registry data demonstrate that invasive mold infections also occur in patients without hematologic disease, including those with solid tumors, particularly in the presence of immunosuppressive therapies [4]. In a meta-analysis of 71 trials including more than 2,000 patients with varied illnesses and glucocorticoid doses, the relative risk of infection was 2 [10]. The associated risk correlates directly with the dose and duration of treatment [10]. Doses as low as prednisone 16 mg/day for eight weeks have been shown to increase the risk of opportunistic infection [10]. Hypercortisolemic patients, including those with exogenous Cushing syndrome, are at even greater risk [10]. In Case 1, the patient had received dexamethasone 4 mg daily (equivalent to 26.7 mg of prednisone) for approximately eight months and had a known diagnosis of exogenous Cushing syndrome before admission, prompting an attempted steroid taper. Therefore, he had a substantially increased risk of opportunistic infections related to corticosteroid use alone, which was likely a major factor in the progression of pulmonary aspergillosis to fungemia.

Findings favoring pseudofungemia include a single positive culture, growth restricted to one medium, absence of clinical correlation, and negative repeat cultures [2]. In Case 2, the patient was receiving sirolimus, tacrolimus, prednisone, and leflunomide simultaneously, with a supratherapeutic sirolimus level exceeding 30 ng/mL at admission, resulting in profound immunosuppression. However, he was already receiving voriconazole prophylaxis, as recommended by the IDSA guidelines for allogeneic hematopoietic stem cell transplant recipients, particularly those with graft-versus-host disease or receiving high-dose corticosteroids [8]. Only one peripheral blood culture was positive and grew a single colony of *Aspergillus *on one plate. Repeat blood cultures and serum galactomannan were negative. In addition, he had no fever or symptoms consistent with respiratory infection or sepsis. His symptoms were limited to gastrointestinal manifestations consistent with biopsy-proven gastrointestinal graft-versus-host disease. Therefore, although this patient was at high theoretical risk for invasive fungal infection, he was likely protected by appropriate antifungal prophylaxis. This management is consistent with the recommended approach when pseudofungemia is suspected: withhold escalation of antifungal therapy, obtain repeat cultures, and monitor the clinical course [2,8].

Mortality associated with *Aspergillus* fungemia is substantial, ranging from 50% to 100% in previous studies [6,7]. In Case 1, timely antifungal management, including source control through port removal, likely contributed to survival despite advanced underlying malignancy, reinforcing that early identification and species-guided therapy are critical determinants of outcome [1,2]. In Case 2, the absence of clinical, radiographic, and serologic corroboration appropriately precluded escalation beyond established prophylaxis, as supported by the patient's uncomplicated subsequent course. Taken together, these cases reflect two distinct outcomes arising from the same diagnostic challenge in very different clinical contexts. Further large-scale research is needed, as the existing literature is limited by the rarity of this diagnosis and the resulting small number of reported patients. In addition, existing studies frequently pool multiple fungal species together; therefore, research dedicated specifically to *Aspergillus *fungemia is warranted.

## Conclusions

These cases illustrate the range of the diagnostic spectrum that clinicians must navigate when managing immunocompromised patients: one in which *Aspergillus *isolated from blood cultures represents a life-threatening disseminated infection requiring urgent source control and treatment and one in which a positive culture reflects contamination without clinical consequence. The ability to distinguish between these possibilities, using clinical correlation, culture reproducibility, colony burden, imaging, and serologic markers, is central to avoiding both undertreatment of true fungemia and unnecessary escalation of antifungal therapy with its associated toxicities. Prolonged corticosteroid use, indwelling vascular devices, and environmental exposures may predispose patients without classic risk factors to invasive infection and should be routinely assessed. As the population of patients receiving complex immunosuppressive regimens continues to grow, the diagnostic and therapeutic strategies described here will become increasingly relevant, underscoring the need for larger, species-specific studies to better define the optimal management of this uncommon but life-threatening condition.
